# Optimising the reach of mobile health messaging programmes: an analysis of system generated data for the Kilkari programme across 13 states in India

**DOI:** 10.1136/bmjgh-2022-009395

**Published:** 2022-08-08

**Authors:** Diwakar Mohan, Jean Juste Harrisson Bashingwa, Kerry Scott, Salil Arora, Sai Rahul, Nicola Mulder, Sara Chamberlain, Amnesty Elizabeth LeFevre, the Kilkari Impact Evaluation Team, Smisha Agarwal

**Affiliations:** 1 Department of International Health, Johns Hopkins University Bloomberg School of Public Health, Baltimore, Maryland, USA; 2 Medical Research Council/ Wits Rural Public Health and Health Transitions Research Unit (Agincourt), School of Public Health, Faculty of Health Sciences, University of the Witwatersrand, 27 St Andrews Road, Parktown, Johannesburg, 2193, South Africa, Johannesburg, South Africa; 3 National Institute for Theoretical and Computational Sciences (NITheCS), South Africa, Johannesburg, South Africa; 4 Computational Biology Division, Department of Integrative Biomedical Sciences, University of Cape Town, Cape Town, South Africa; 5 Digital, BBC Media Action, New Delhi, India; 6 Beehyv Software Solutions Limited, Hyderabad, Telangana, India; 7 BBC Media Action, Delhi, Delhi NCR, India; 8 Asia, BBC Media Action, London, UK; 9 School of Public Health and Family Medicine, University of Cape Town, Cape Town, South Africa

**Keywords:** Health services research, Maternal health, Prevention strategies

## Abstract

Kilkari is an outbound service that makes weekly, stage-based, prerecorded calls about reproductive, maternal, neonatal and child health directly to families’ mobile phones, starting from the second trimester of pregnancy and until the child is 1 year old. Since its initiation in 2012–2013, Kilkari has scaled to 13 states across India. In this analysis article, we explored the subscriber’s journey from entry to programme to engagement with calls. Data sources included call data records and household survey data from the 2015 National Family Health Survey. In 2018, of the 13.6 million records received by MOTECH, the technology platform that powers Kilkari, 9.5 million (~70%) were rejected and 4.1 million new subscribers were created. Overall, 21% of pregnant women across 13 states were covered by the programme in 2018, with West Bengal and Himachal Pradesh reaching a coverage of over 50%. Among new subscriptions in 2018, 63% were subscribed during pregnancy and 37% after childbirth. Of these, over 80% were ever reached by Kilkari calls and 39% retained in the programme. The main causes for deactivation of subscribers from the system were low listenership and calls going unanswered for six continuous weeks. Globally, Kilkari is the largest maternal mobile messaging programme of its kind in terms of number of subscribers but the coverage among pregnant women remains low. While call reach appears to be on the higher side, subscriber retention is low; this highlights broader challenges with providing mobile health services at scale across India.

**Summary box**

- Direct to Beneficiary (D2B) programs are among the few digital health programs to scale widely in a range of low and middle income countries where the majority of maternal and child deaths occur.
- Kilkari, an outbound audio messaging service which provides women and their families in India with health information, is the world’s largest D2B program.
- The variable quality of data in government tracking registries underpins low (21%) subscriber coverage of pregnant women at the population level.
- Despite low population level coverage, over 80% of subscribers are reached by the program and greater than one-third remained enrolled in the program until the completion of messages.
- Study findings suggest that to improve population level coverage of Kilkari would require addressing limitations in the quality of data in tracking registries, including updating phone numbers with greater frequency. However, if women are subscribed, they are reached with health information content and tend to remain engaged in the program through completion.

## Introduction

Digital health encompasses the subsectors of health information technology, mobile health (mHealth), electronic health, telehealth and telemedicine. Evidence gathering on the effectiveness of digital health solutions is a growing field.^1^ Mobile phones are becoming ubiquitous and, increasingly, an important tool in global health programmes.^2–4^ Mobile phones have the potential to connect clients with healthcare providers, optimise data collection, provide new avenues of delivering information and facilitate healthcare worker training and communication.^4–8^ Their increasing use as a channel for the delivery of health information content has shown some promise in bolstering health behaviours and demand for timely and appropriate health services in a range of settings.^9 10^

Kilkari (the Hindi word for a baby’s gurgle) is an outbound service that delivers weekly, gestational age appropriate interactive voice response audio messages about pregnancy, childbirth and childcare directly to families on their mobile phones, starting from the second trimester of pregnancy until the child is 1 year old.^11^ Established by BBC Media Action and the Indian Ministry of Health and Family Welfare in 2013, Kilkari has scaled to over 13 states and is estimated to have over 10 million subscribers. The programme draws information on mobile phone numbers and gestational age or the baby’s date of birth from government electronic tracking registries. Depending on timing of enrolment during pregnancy or following childbirth, subscribers may receive up to 72 weekly audio messages. Additional details on the programme are available elsewhere.^11^

Evidence on the impact of Kilkari is emerging,^12^ along with details on subscribers’ perceptions of content^13^ and exposure.^10 14^ However, little is known about the programme’s eligibility, reach and retention at scale across 13 states. Kilkari’s subscriber base is drawn from data in government tracking registries captured by frontline health workers (FLHWs) including Accredited Social Health Activists and Auxiliary Nurse Midwives. Data recorded in print registers are uploaded into electronic records, and ultimately, ingested into the Kilkari programme’s platform called MOTECH. The quality of registry data helps to determine the proportion of women at population level eligible for the programme (coverage), and the programme’s reach defined in terms of the gestational timing of first call and alignment of those calls with the target schedule. Retention is defined by the proportion of beneficiaries that remain in the programme through completion (1-year post partum) and is assessed by exploring deactivation trends.

Improved understanding of Kilkari’s eligibility, reach and retention aims to shed light on differences in programme performance across geographical areas. As programmes scale, they may undergo a ‘voltage drop’ in reach and impact attributed in part to the changes in the programme design and implementation required to scale, including strategies to recruit patients, promote the programme and update beneficiary information (eg, phone numbers, date of birth) to ensure continuity in access to messages.^15 16^ Understanding of the users’ journey from eligibility and entry into the programme, through to completion, will help to identify key inflection points where programmatic strategies can be developed to intervene, bolster programme performance and beneficiary engagement and ultimately, improve impact.

**Figure 1:**
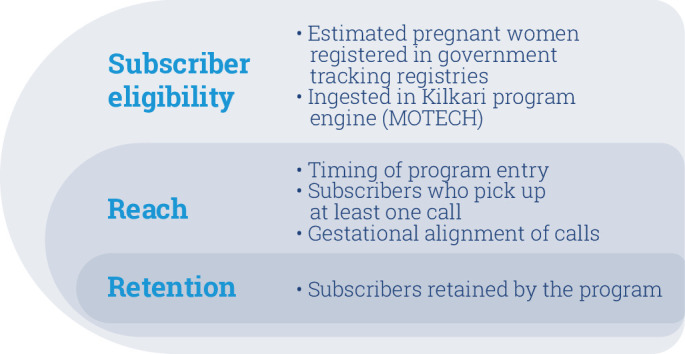
Framework conceptualising eligibility, reach and retention in the Kilkari programme.

In this analysis article, we draw from system generated data on subscribers across 13 states who entered the Kilkari programme in 2018 to explore eligibility for subscription, reach and retention (figure 1). To determine eligibility for subscription, we start by estimating the proportion of pregnant women contained within the government tracking registries which drives Kilkari’s coverage at a population level. We next explore the timing of when subscribers are first called during pregnancy or postpartum; a factor which establishes the window of exposure to Kilkari calls. Among subscribers who answer at least one call (and thus are ‘reached’) during pregnancy, we determine the frequency of date of birth updates which serve to ensure that calls are aligned with women’s gestational/postpartum age. We conclude by assessing retention in the programme and ascertaining the reasons for deactivation. Overall findings from this analysis aim to illustrate the subscriber’s journey from entry in the programme and the timing of the first call to completion at 12 months post partum. By mapping drop-offs at each point along this journey, we aim to catalyse discourse on how to maximise future coverage, reach and retention.

## Subscriber eligibility

### Variable capture of pregnancies in government tracking registries: an average of 21% of pregnancies across the 13 states were recorded in government tracking registries

Data (phone numbers, last menstrual period (LMPs), and date of the child’s birth) drawn from government tracking registries and ingested into MOTECH comprise the sampling frame for Kilkari. At the time of ingestion, a series of validation checks are undertaken to ‘clean’ the data. The validation checks ensure that mobile numbers are not duplicates, have the correct number of digits and contain no special characters. Records with missing or invalid mandatory data fields are rejected. Those records that pass through these validation checks go on to then receive Kilkari calls.

**Figure 2:**
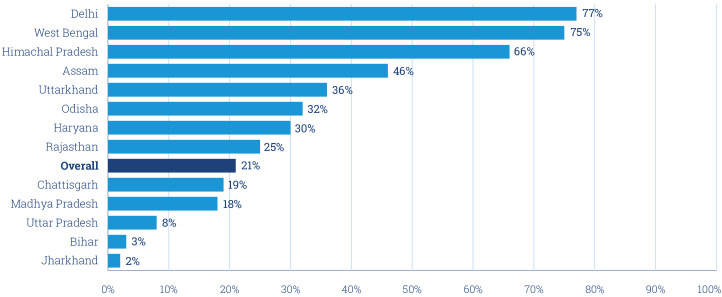
Percentage of pregnant women (modelled) with a record in the Kilkari database for 2018.

For the calendar year 2018, nearly 13.6 million records were received from government tracking registries; 70% of these were rejected during validation checks. The leading reasons for rejection included invalid LMPs or date of birth (46%) or duplicate phone numbers (40%) (online supplemental figure 1). If we estimate the proportion of pregnant women at a population level (box 1), we see that the database only captures 21% of pregnancies—although this varied widely from 77% in Delhi to 3% in Bihar (figure 2). Ultimately, 4.1 million mother subscribers were created in 2018, with an average rate of 300 000–400 000 subscriptions created every month (online supplemental figure 2). Collectively, these findings indicate that if population level coverage of Kilkari is to increase, improvements are needed in (a) women’s mobile phone access (currently estimated at 47·8%); and (b) the accuracy and completeness of data in government tracking registries. Elsewhere we outline recommendations for addressing the latter.^14 17^

10.1136/bmjgh-2022-009395.supp1 Supplementary data

Box 1 **Modelling expected pregnancies in 1 year**

**Step 1: Estimate the mid-year population**

The census data for 2011 was used to calculate the mid-year population for 2017 using an annual growth rate of 1.1%. The following formula was used to calculate

***Mid-year population 2011 × (AGRˆTime) = Mid-year population 2017***

Sources

- Mid-year population 2011 for each state – http://www.censusindia.gov.in/2011census/population_enumeration.html.
- AGR (annual growth rate) – 1.1% https://data.worldbank.org/indicator/SP.POP.GROW.
- Time – 6 years (2017 minus 2011).

**Step 2: Estimate the expected number of pregnancies**

Crude birth rates was from the 2016 estimates provided by the Sample Registration System of the Indian census division were used to calculate the estimated number of pregnancies.

***Mid-year population 2017 × Crude birth rate × (1+P) = Expected number of pregnancies***

Sources

- Crude birth rate for each state – https://data.gov.in/catalog/crude-birth-rate-india.
- P – A factor accounting for pregnancy losses was assumed to be 10% for all states.

## Reach

### Timing of programme entry: 37% of subscriptions occurred after the birth of the child

**Figure 3:**
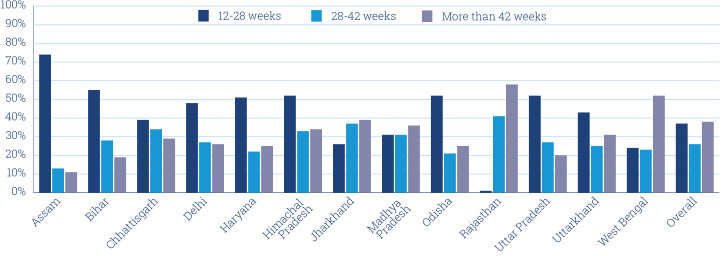
Timing of subscriptions in the Kilkari programme in 2018 based on the stage of pregnancy for selected states. Black bars denote the proportion of subscribers registered during the second or third trimester of pregnancy. White bars denote the proportion of subscribed 0–6 months post partum and blue bars 6–12 months post partum.

The 72 weekly Kilkari calls are intended to span from the 12th week of pregnancy through 1-year post partum. However, for the maximum period of exposure to occur, pregnancies need to be identified by FLHWs early and the relevant information on phone numbers and LMP captured into printed registers, uploaded into electronic records and ingested into the Kilkari programme engine. In 2018, 37% of subscriptions occurred after the birth of the child—meaning they miss the first 24 weeks of Kilkari calls. States varied widely in their ability to bring in subscribers in a timely manner. In Assam, three-quarters of subscriptions started between 12 and 28 weeks of pregnancy. However, in Rajasthan the majority of the subscriptions occurred after 42 weeks from the date of the LMP and thus were considered as being after delivery (figure 3). Such delays may be the result of multiple factors including poor outreach by FLHWs, complexities in collection of relevant information and the timeliness of data entry into electronic portals.^14^

### Eighty per cent of subscribers who receive calls answer at least one of the first six call attempts made

**Figure 4:**
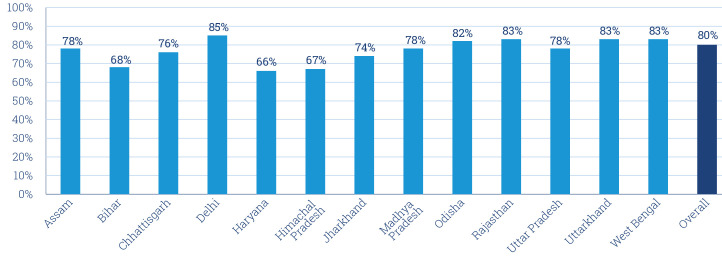
Percentage of subscriptions in the Kilkari programme who answered a call at least once across states.

Of these subscribers called, only those who answered at least one of the first six calls made to them were considered as being reached by the programme. Across the 13 states, 80% of subscribers are reached—that is, at least one of the first six calls are answered (figure 4). The percentage reached is consistent across states ranging from 66% to 85%. Subscribers who do not answer one of the first six calls made to them are deactivated to free up infrastructure space needed to enrol new subscribers.

Among calls answered, over 63% of subscribers listened to 50% or more of the cumulative content of Kilkari calls received. Elsewhere we explore exposure to Kilkari calls and their content in greater detail.^18^ In brief, the audio messages delivered as part of the Kilkari programme are divided into three segments: (1) introduction to the topic; (2) a series of knowledge content and calls to action and; (3) a summation. For practical purposes, listening to the first 50% of the duration of a call would have covered the most important parts of the call. Among calls answered, over 63% of subscribers listened to 50% or more of the cumulative content of Kilkari calls answered, and almost half listened to more than 75%.

### Gestational alignment of calls: nearly two-thirds of subscribers enrolled during pregnancy did not have date of birth updates

Among subscribers registered during pregnancy, subscriptions are expected to be updated with the pregnancy outcome, including the date of birth for the child(ren) or details of adverse outcomes, including miscarriages or stillbirths. When the record is updated with the date of birth, the programme resets the delivery of calls to align with the appropriate age of the child. In government tracking registries, records are expected to be updated by the FLHW as soon as the delivery occurs. However, in practice, the intimation of a birth might take many weeks, if not months. In 2018, among subscribers enrolled during pregnancy, only 31% had their records updated to include the child’s date of birth. In practice this means that 69% of those entering during pregnancy may not be receiving the Kilkari call aligned with their child’s age. In cases where updates occurred, the timing of these updates varied considerably (online supplemental figure 3). In Rajasthan, date of birth updates occurred for 50% of existing subscribers within the first month post partum while other states had up to 40% of their existing subscriptions updated 3 months or more after the birth of the child. Kilkari health content in areas such as breastfeeding and essential newborn care are most likely to be impacted.

For a messaging programme working at scale, Kilkari calls are received by a high proportion of enrolled subscribers though this does not guarantee that all those who answer are pregnant or postpartum women. There is much room for improvement in quality such as getting subscribers into the programme on time (early in pregnancy) and adjusting messaging to gestational age or age of child (by updating birth information).

## Subscriber retention

Kilkari subscribers may be deactivated from receiving Kilkari calls and thus leave the programme. There are four reasons why this may occur: 1)subscribers could unsubscribe themselves, 2)manually deactivated for failing to answer calls for six consecutive weeks, 3)manually deactivated due to low levels of listening (defined as less than 25% of a message for six messages) or 4)adverse pregnancy outcomes. Among those subscribers reached, 39% remained in the programme through 12 months post partum. The remaining 61% were deactivated.

**Figure 5:**
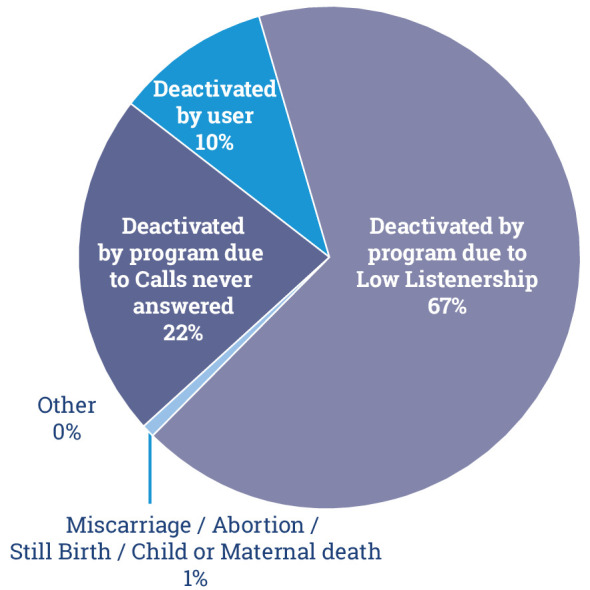
Reasons for the deactivation of subscribers from the Kilkari programme in 2018.

The leading reason for deactivations was low listenership (67%), followed by not answering calls (22%), deactivation by subscribers (10%) and adverse pregnancy outcomes (<1%) (figure 5). Established thresholds for deactivating subscribers due to low listenership have varied from 12 weeks to 6 weeks over the life of the programme to accommodate demand for new subscriptions and infrastructure constraints. In 2018, subscribers who listened to <25% of the content on every call for 6 weeks were considered low listeners and subsequently deactivated. Deactivations attributed to low listening and call answer rates may be a result of SIM churn. A market survey from 2010 showed that nearly 40% of subscribers report changing their mobile numbers every 2 years while one-third report using multiple SIMs either with dual or single SIM capable phones.^19^ Low engagement, evidenced by low listening, appears to be the primary reason for subscriber deactivation. The high SIM churn and poor quality of data on subscriber phone numbers offers the possibility that the listener receiving the phone call may not be a pregnant or postpartum woman, again pressing home the need for better data and improving mobile phone access for women.

### Implications for future programmes

Across the 13 states where implementation is underway, approximately 22.1 million pregnant women are eligible for Kilkari; of these only 4.7 million (21%) were reached—defined as receiving and answering at least one call. The greatest drop-offs occur at the step of making it into the government’s pregnancy tracking registries. To increase eligibility and reach of the Kilkari, the quality of data in government tracking registries needs to be improved. Improvements in the quality of registry data would additionally help to bolster the timeliness of programme entry and the overall window of exposure.

The current programme passively subscribes beneficiaries based on the data captured in government tracking registries. Elsewhere globally, similar programmes in South Africa have used FLHWs to explain the programme and capture informed consent prior to initiating message delivery.^20^ While this approach at scale in India may be prohibitively resource intensive, efforts could be made to improve FLHW awareness and promotion and create systems for ‘opting-in’ beneficiaries and enabling the updating of relevant data fields including phone numbers and the child’s date of birth. These modifications to the programme might serve to improve beneficiary engagement, exposure and in turn, the impact of calls.

A recently published randomised controlled trial showed that exposure to Kilkari was significantly associated with improvements in a few important health practices, including the use of reversible contraceptive methods with a greater impact the poorest.^10^ These improvements in coverage of life saving interventions, though small in magnitude, have the potential to save many lives when translated across millions.

The industry of direct to consumer (direct to beneficiary) services is growing rapidly and will benefit greatly from segmentation and differential targeting of consumers (or beneficiaries). These require access to better quality data to feed data hungry algorithms and testing of different strategies for retention (eg, A/B testing). Such programme level changes may also need to be accompanied by changes at the macro level including improved mobile phone access (particularly to young women), telecommunications upgrades to strengthen network connectivity and stabilising SIM churn.

## Conclusions

Globally, Kilkari is the largest direct to beneficiary mHealth programme in the world. Improving the quality of data in government tracking registries would not only bolster the total number of pregnant women eligible to receive Kilkari, but also improve the timeliness of programme entry and maximise the window of exposure. Efforts to improve beneficiary and FLHW awareness of Kilkari, create systems for ‘opting-in’ beneficiaries and enable the updating of phone numbers may improve programme reach, subsequent beneficiary engagement with calls and, in turn, their impact. In the absence of these features, the Kilkari programme’s reach and potential impact has yet to be fully realised.

## Data Availability

Data may be obtained from a third party and are not publicly available. The data for the present analysis was provided by the Ministry of Health & Family Welfare, Government of India.
