# Supplementary material for: Optimising the reach of mobile health messaging programmes: an analysis of system generated data for the Kilkari programme across 13 states in India
Source: BMJ Glob Health. 2022 Aug 8;6(Suppl 5):e009395. doi: 10.1136/bmjgh-2022-009395 (PMC9366343; doi:10.1136/bmjgh-2022-009395)

Supplementary Figure 1. Reasons for rejection of records from ingestion into Kilkari program engine for 2018

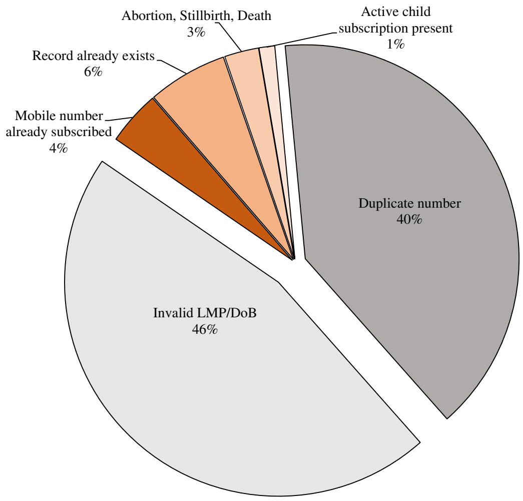

Supplementary Figure 2. Monthly subscriptions across different states created by pregnant women in the Kilkari program for 2018

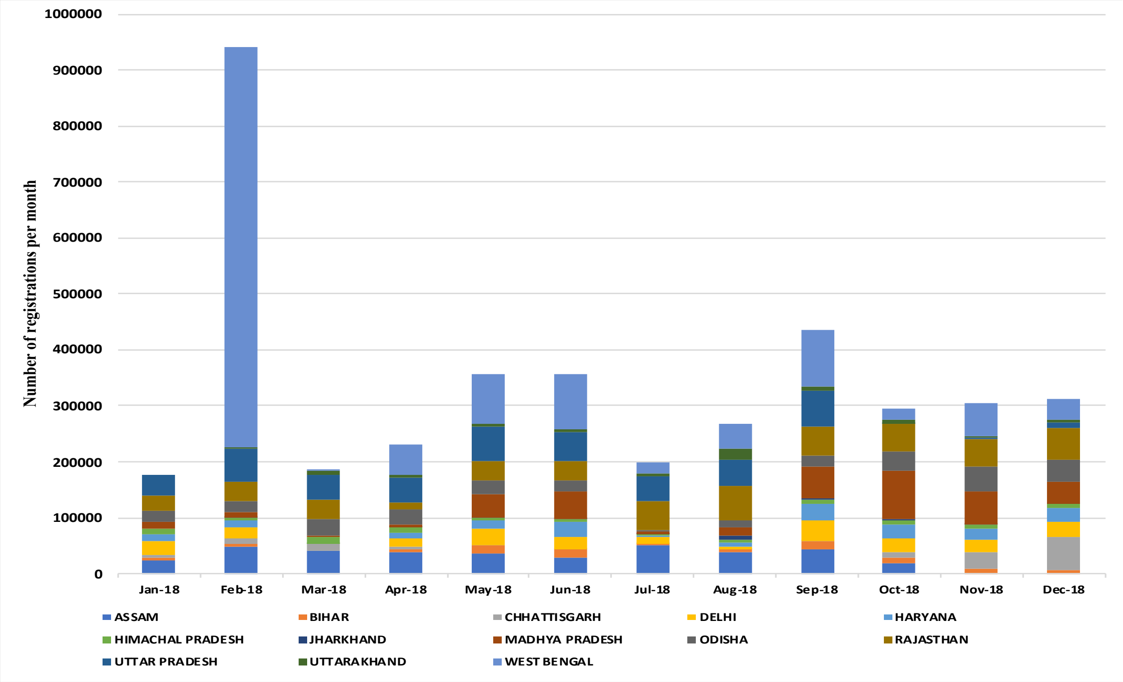

Supplementary Figure 3 - Percentage of subscribers enrolled during pregnancy in 2018 with an updated child's date of birth

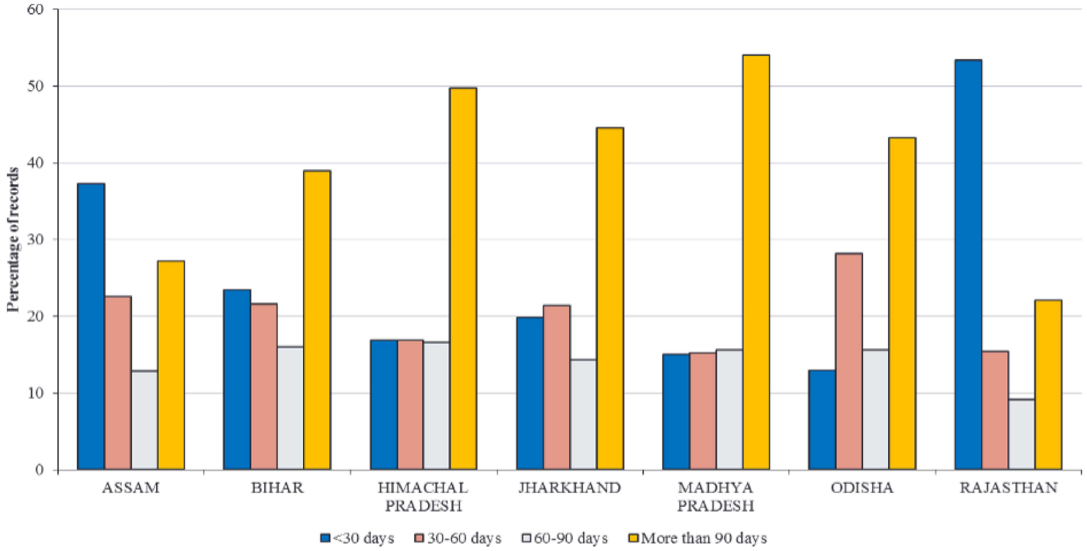

Supplement: Supplementary data [file bmjgh-2022-009395supp001.pdf]
